# Calcitriol Inhibits HCV Infection via Blockade of Activation of PPAR and Interference with Endoplasmic Reticulum-Associated Degradation

**DOI:** 10.3390/v10020057

**Published:** 2018-01-30

**Authors:** Yu-Min Lin, Hung-Yu Sun, Wen-Tai Chiu, Hui-Chen Su, Yu-Chieh Chien, Lee-Won Chong, Hung-Chuen Chang, Chyi-Huey Bai, Kung-Chia Young, Chiung-Wen Tsao

**Affiliations:** 1 Department of Gastroenterology, Shin Kong Wu Ho-Su Memorial Hospital, Taipei 11101, Taiwan; M001063@ms.skh.org.tw (Y.-M.L.); M002291@ms.skh.org.tw (L.-W.C.); twliverhcc@gmail.com (H.-C.C.); 2 Department of Medical Laboratory Science and Biotechnology, College of Medicine, National Cheng Kung University, Tainan 70101, Taiwan; s5893149@gmail.com (H.-Y.S.); magchien@hotmail.com (Y.-C.C.); t7908077@mail.ncku.edu.tw (K.-C.Y.); 3 Department of Biomedical Engineering, College of Engineering, National Cheng Kung University, Tainan 70101, Taiwan; wtchiu@mail.ncku.edu.tw; 4 Department of Pharmacy, Chi-Mei Medical Center, Tainan 71004, Taiwan; michael.maggil@gmail.com; 5 Department of Long Term Care, Chung Hwa University of Medical Technology, Tainan 71703, Taiwan; 6 Department of Public Health, College of Medicine, Taipei Medical University, Taipei 11031, Taiwan; baich@ms4.hinet.net

**Keywords:** calcitriol, hepatitis C virus infection, peroxisome proliferator-activated receptor, endoplasmic reticulum-associated degradation, nitrative stress

## Abstract

Vitamin D has been identified as an innate anti-hepatitis C virus (HCV) agent but the possible mechanisms for this issue remain unclear. Here, we clarified the mechanisms of calcitriol-mediated inhibition of HCV infection. Calcitriol partially inhibited HCV infection, nitric oxide (NO) release and lipid accumulation in Huh7.5 human hepatoma cells via the activation of vitamin D receptor (VDR). When cells were pretreated with the activators of peroxisome proliferator-activated receptor (PPAR)-α (Wy14643) and -γ (Ly171883), the calcitriol-mediated HCV suppression was reversed. Otherwise, three individual stimulators of PPAR-α/β/γ blocked the activation of VDR. PPAR-β (linoleic acid) reversed the inhibition of NO release, whereas PPAR-γ (Ly171883) reversed the inhibitions of NO release and lipid accumulation in the presence of calcitriol. The calcitriol-mediated viral suppression, inhibition of NO release and activation of VDR were partially blocked by an inhibitor of endoplasmic reticulum-associated degradation (ERAD), kifunensine. Furthermore, calcitriol blocked the HCV-induced expressions of apolipoprotein J and 78 kDa glucose-regulated protein, which was restored by pretreatment of kifunensine. These results indicated that the calcitriol-mediated HCV suppression was associated with the activation of VDR, interference with ERAD process, as well as blockades of PPAR, lipid accumulation and nitrative stress.

## 1. Introduction

Vitamin D is essential to regulation of calcium homeostasis, is involved in cell proliferation and differentiation and exerts an immunomodulatory and anti-inflammatory property. Vitamin D is sourced from skin with ultraviolet B exposure and from dietary intake to be synthesized and then partially stored in the adipocytes. Alternatively, it produces in part 25-hydroxyvitamin D via hepatic 25-hydroxylation, which is sent into circulation, attached to vitamin D binding protein and transported to target tissues. 25-Hydroxyvitamin D is also hydroxylated by 25-hydroxyvitamin D3 1-α-hydroxylase (CYP27B1) in the proximal tubules of the kidney to form the biologically active form, 1,25-dihydroxyvitamin D3 (calcitriol), which it further acts on vitamin D receptor (VDR) to regulate biological functions [1,2].

Vitamin D deficiency participates in pathological mechanisms involved in diabetes mellitus, cardiovascular diseases, mood disorders, declining cognitive functions and increased risk of infections [1,2]. Several studies have recently focused on vitamin D deficiency to be associated with the development of chronic liver diseases, including non-alcoholic fatty liver disease and hepatitis C virus (HCV) infection with a lower probability of a sustained virological response (SVR) to interferon-α (IFN-α) regimen [3,4]. However, some findings that the intervention of vitamin D improve the anti-HCV of IFN-α to achieve a SVR have been shown to be debatable [5]. Whether the vitamin D deficiency acts as a consequence of disease or as a contributor to the dysfunction remains obscure [3,4,5].

Interestingly, Huh7.5 hepatoma cells also express CYP27B1 and 1,25-dihydroxyvitamin D3 24-hydroxylase (CYP24A1), which converts vitamin D3 to calcitriol, afterwards to calcitroic acid, indicating that hepatocytes comprise the entire apparatus for vitamin D metabolism and activity. Vitamin D, 25-hydroxyvitamin D, or calcitriol also inhibits HCV [6,7]. Thus, vitamin D and its metabolites have also been acknowledged as an innate antiviral agent [6]. Nevertheless, the precisely possible mechanisms for calcitriol-mediated inhibition of HCV infection are still unclear.

The main focus of interest at the pathogenesis of HCV infection includes virus infection, lipid metabolism [8], oxidative and nitrative stress [9,10,11], endoplasmic reticulum (ER) stress [11,12,13], ER-associated degradation (ERAD) pathway [14] and host anti-viral signals [11,15]. Previous studies also indicate that vitamin D or calcitriol regulates lipid metabolism [16,17,18,19,20], affects apolipoprotein (apo) and cholesterol levels [21,22,23,24], prevents nitrative stress [25] and ER stress [26,27,28] and negatively modulates peroxisome proliferator-activated receptor (PPAR)-related signals [17,20,27].

Therefore, the mechanisms of calcitriol-mediated inhibition of HCV infection are further clarified in the present study, especially via the regulations of PPAR and ERAD. Nitrative stress, lipid accumulation with altered apo expressions (e.g., apoJ), ER stress sensors (e.g., 78 kDa glucose-regulated protein/binding immunoglobulin protein, Grp78/Bip) and the activation of VDR, were also investigated following the HCV-infected Huh7.5 human hepatoma cells that had been pretreated with the PPAR activators or ERAD blocker, then cultured with calcitriol.

## 2. Materials and Methods

### 2.1. Chemicals

Calcitriol and kifunensine were purchased from Tocris Bioscience (Ellisville, MO, USA). Linoleic acid, Ly171883 and Wy14643 were purchased from Sigma-Aldrich (St. Louis, MO, USA).

### 2.2. Antibodies

Mouse monoclonal antibody [C7-50] to HCV core 1b (catalog No. ab2740), mouse monoclonal antibody [8 G-2] to HCV non-structural (NS) protein NS3 (catalog No. ab65407), rabbit polyclonal antibody to apoJ/clusterin [synthetic peptide corresponding to human apoJ/clusterin (C terminal)] (species: human; catalog No. ab69644) and rabbit monoclonal [EPR4041(2)] to Grp78/Bip (species: mouse and human; catalog No. ab108615) were purchased from Abcam (Cambridge, UK). Monoclonal anti-β-actin antibody [AC-15] (species: human, bovine, sheep, pig, rabbit, cat, dog, mouse, rat, guinea pig, chicken, carp; catalog No. A5441) produced in mouse was purchased from Cell Signaling Technology, Inc. (Beverly, MA, USA). Monoclonal antibody [9A7] to VDR (species: dog, avian, rat, non-human primate, hamster, mouse, human, chicken; catalog No. MA1710) was purchased from Thermo Fisher Scientific Inc. (Rockford, IL, USA).

### 2.3. Cell Culture

The Huh7.5 cells that carry NS3/4A protease-based secreted alkaline phosphatase (SEAP) reporter were cultured in Dulbecco’s Modified Eagle’s Medium (Hyclone, South Logan, UT, USA) containing 2 μg/mL blasticidin (Life Technologies, Grand Island, NY, USA).

### 2.4. HCV Virus Stock

The HCV virus stock was obtained by concentrating the JFH-1 virion-containing supernatant through polyethylene glycol-8000 (Sigma-Aldrich) and its titer was determined via the immunofluorescence assay of HCV core-positive foci [29]. This prepared virus stock was later used for the infection of naïve Huh7.5 cells at the multiplicity of infection (MOI) of 0.01.

### 2.5. Evaluation of HCV Infection Using NS 3/4A Protease-Based SEAP Reporter Assay

The SEAP activity in the culture media was evaluated using a Phospha-Light assay kit (Invitrogen, Carlsbad, CA, USA) as described in the previous study [29].

### 2.6. Cell Viability

A commercial kit for 3-(4,5-dimethylthiazol-2-yl)-5-(3-carboxymethoxyphenyl)-2-(4-sulfophenyl)-2*H*-tetrazolium (MTS) assay (Promega, Madison, WI, USA) was applied to examine the cell viability via a measurement of Epoch Microplate Spectrophotometer (BioTek, Winooski, VT, USA) at 490 nm.

### 2.7. PPAR Response Element (PPRE) Activation

The PPREx3Luc plasmid contained three tandem repeats of the PPRE upstream of firefly luciferase reporter gene was constructed previously [30]. Huh7.5 cells comprising PPREx3Luc were treated with calcitriol for 6 h following the HCV infection at MOI 0.01 for 18 h. After the collection of cell lysates, the activity of luciferase reporter was examined via a luciferase reporter assay (Promega, Madison, WI, USA).

### 2.8. Western Blot Analysis

Cell lysates were collected at indicated times using lysis buffer containing 1% Triton X-100, 50 mM Tris (pH 7.5), 10 mM EDTA, 0.02% NaN_3_ and protease inhibitor cocktail (Sigma-Aldrich). After being centrifuged for 10 min at 13,000 rpm, 4 °C, proteins in the collected supernatant were quantified and separated using 10% SDS-polyacrylamide gel electrophoresis and they were transferred to a polyvinylidene difluoride membrane (Millipore, Billerica, MA, USA). The membrane was blocked with 5% skim milk in TBS-T (10 mM Tris, 150 mM NaCl and 0.05% Tween 20, pH 7.6) for 1 h at room temperature and probed with primary antibodies at 4 °C overnight. Horseradish peroxidase-conjugated secondary antibodies were applied to the membrane for 1 h at room temperature after being washed with TBS-T. The protein expressions were visualized via enhanced chemiluminescence reagent (PerkinElmer, Boston, MA, USA) and analyzed with VisionWorks^®^LS software (version 8, Upland, CA, USA). β-actin acted as the internal control.

### 2.9. Immunofluorescence Analysis

Cells were treated with calcitriol alone and/or pretreatment with individual PPAR activator or ERAD inhibitor for 5 days in a 24-well plate. Then, cells were sub-cultured and seeded (1.5 × 10^4^) to 4-well Millicell EZ^®^ slide (Merck Millipore, Burlington, MA, USA) during same medium condition for 1 day. The cells were harvested, fixed with 4% paraformaldehyde for 20 min at room temperature, permeabilized with 0.2% Triton X-100 for 10 min and 5% bovine serum albumin/phosphate buffered saline for 30 min at room temperature and stained with primary antibodies against the core, VDR, apoJ and Grp78 overnight. Secondary antibodies, including Alexa Fluor 594-/488-conjugated donkey anti-rat IgG, 647-conjugated donkey anti-mouse/goat anti-rabbit IgG, 488-conjugated goat anti-mouse IgG, and 568-conjugated goat anti-rabbit IgG, were used the next day and stained for 1 h at room temperature at 70 rpm. The nucleus was stained with 4,6-diamidino-2-phenylindole (DAPI, Sigma-Aldrich) for 2 min at room temperature at 70 rpm. After being carefully washed, these samples on glass slides were mounted and observed under confocal laser scanning microscope (Olympus FluoView^TM^, FV1000, Shinjuku-ku, Tokyo, Japan).

### 2.10. Determination of Nitric Oxide (NO) Release

The accumulated levels of nitrite in supernatant were determined for NO production. In short, collected supernatant was reacted with Griess reagent (1% sulphanilamide in 5% H_3_PO_4_ and 0.1% naphthylethylenediamine dihydrochloride) at a ratio of 1:1 for 5 min at room temperature and measured at a wavelength of 550 nm. The nitrite concentration was determined according to a standard curve of sodium nitrite via an ELISA software (Softmax Pro; Molecular Devices, Version 5.4.1, Sunnyvale, CA, USA).

### 2.11. Lipid Accumulation

Cells were fixed with 7.5% formaldehyde, stained with 0.3% oil red O solution (Sigma-Aldrich) and counterstained with hematoxylin for 15 min. After being rinsed with sterilized water, the sample was air-dried more than 24 h before using 4% IGEPAL-CA-630 (Sigma-Aldrich) to dissolve the oil red O. The OD value was measured by Epoch Microplate Spectrophotometer (BioTek) at 520 nm.

### 2.12. Statistical Analysis

Continuous variables were expressed as the mean ± standard deviation (SD). Variables were compared by using the student’s *t*-test and one-way ANOVA. Statistical significance was set at *p* < 0.05.

## 3. Results

### 3.1. Calcitriol Inhibited HCV Infection via the Activation of VDR

**Figure 1:**
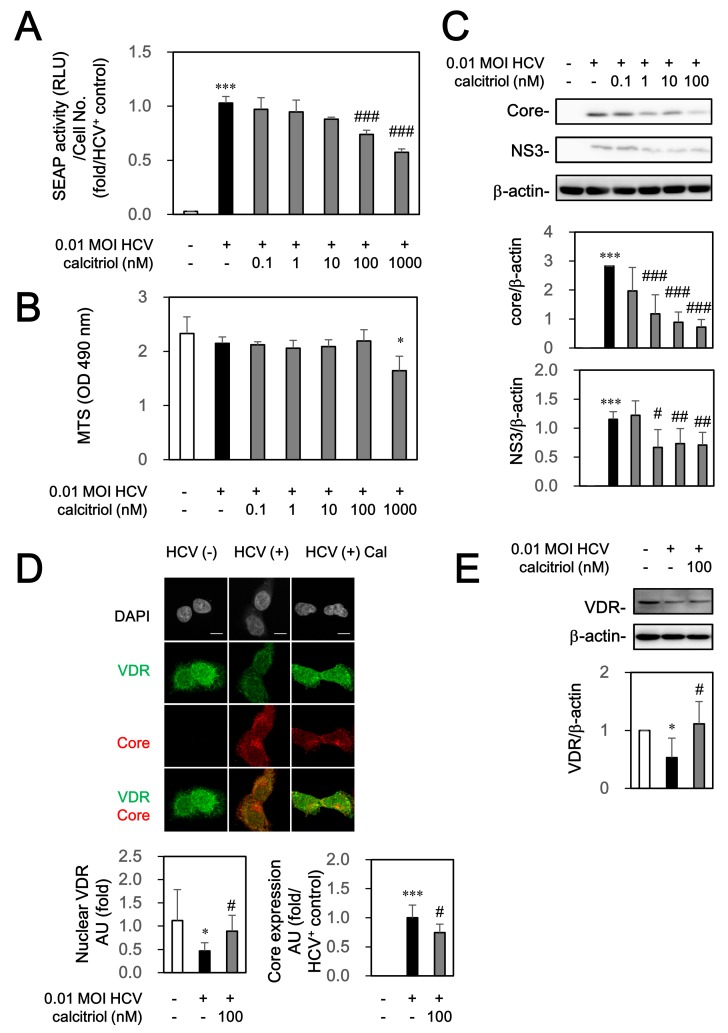
Effects of calcitriol on SEAP activity (**A**); cell viability (**B**) and protein expressions of NS3 (**C**); core (**C**,**D**) and VDR (**D**,**E**). (**A**) Huh7.5-SEAP cells (8 × 10^3^) were infected with HCV (MOI 0.01) and treated with calcitriol (Cal, 0.1–1000 nM) in a 96-well plate for 6 days. The supernatant was subsequently collected for determining the SEAP activity and this activity was normalized with the number of cells as a reflection of the quantitative evaluation of HCV infection; (**B**) Cells were then examined by MTS assay kit; (**C**,**E**) Cell lysates (7 × 10^5^) were collected and the core, NS3, VDR and β-actin levels were detected by Western blot; (**D**) Cells (1.5 × 10^4^) were treated with 100 nM of calcitriol for 6 days in a 24-well plate, fixed with 1% paraformaldehyde, stained with core and VDR antibodies and counterstained with DAPI. After staining, these cells were mounted on glass slides and observed under confocal laser scanning microscope. Scale bar, 10 μM. The arbitrary units (AU) of immunofluorescence intensity were assessed using Image J. Data are expressed as mean ± SD obtained from three individual experiments. * *p* < 0.05 and *** *p* < 0.01 vs. the medium control group without HCV infection. ^#^ *p* < 0.05 and ^##^ *p* < 0.01 and ^###^ *p* < 0.001 vs. the HCV-infected Huh 7.5-SEAP group. The symbol (–) and (+) referred to the cells without and with HCV infection, respectively.

The Huh7.5-SEAP cells infected with HCV at MOI of 0.01 were treated with various concentrations of calcitriol for 6 days to examine whether calcitriol could directly affect HCV. The supernatant was subsequently collected for determining the SEAP activity and this activity was normalized with the number of cells as a reflection of the quantitative evaluation of HCV infection. We found that calcitriol inhibited partially HCV infection at 0.1–1000 nM (Figure 1A). Calcitriol (<1000 nM) did not cause cell cytotoxicity, determined by MTS cell proliferation assay (Figure 1B). Lower viral core and NS3 levels were also detected by calcitriol treatment (Figure 1C). HCV could down-regulate VDR, whose activation was partially restored after treatment of calcitriol, determined by immunofluorescence analysis (Figure 1D) and western blot (Figure 1E). Furthermore, HCV could inhibit translocation of VDR to nucleus, quantified by Image J (Figure 1D).

### 3.2. Calcitriol Inhibition of HCV Infection via the PPAR and ERAD Dependent Pathways

**Figure 2:**
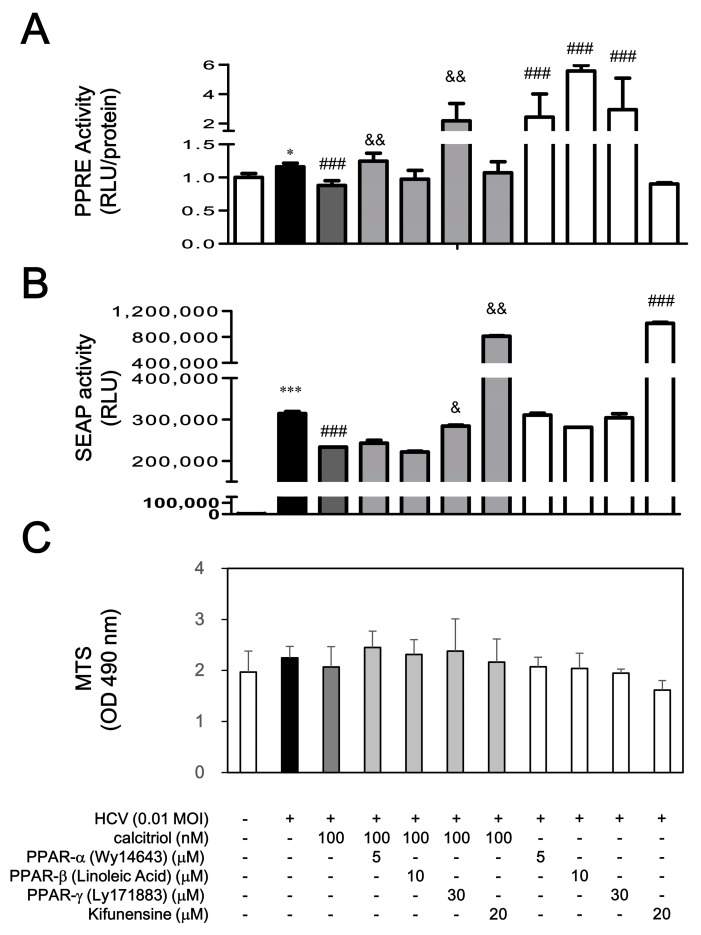
Effects of PPAR stimulators and ERAD inhibitor on calcitriol-mediated PPRE luciferase activity (**A**); SEAP activity (**B**) and cell viability (**C**) in HCV infection. (**A**) Cells transfected with PPREx3Luc (4 × 10^4^) were pre-incubated with 5 μM of Wy14643, 10 μM of linoleic acid, 30 μM of Ly171883, or 20 μM of kifunensine for 1 h and treated with 100 nM of calcitriol for 6 h in a 24-well plate. Subsequently, cells were infected with HCV at MOI 0.01 for 18 h, lysed and assayed via a luciferase reporter assay system; (**B**) HCV-infected Huh7.5-SEAP cells (8 × 10^3^) were pre-incubated with 5 μM of Wy14643, 10 μM of linoleic acid, 30 μM of Ly171883, or 20 μM of kifunensine for 1 h and treated with 100 nM of calcitriol for 6 h in a 96-well plate for 6 days. The cell supernatants were collected for determining SEAP activity; (**C**) The cells were then examined with the MTS assay. Data are expressed as mean ± SD obtained from three individual cultures. * *p* < 0.05 and *** *p* < 0.001 vs. the Huh7.5 cells without HCV infection. ^###^ *p* < 0.001 vs. the HCV-infected Huh7.5-SEAP group. ^&^ *p* < 0.05 and ^&&^ *p* < 0.01 vs. the calcitriol-treated group. The symbol (–) and (+) referred to the cells without and with HCV infection, respectively.

**Figure 3:**
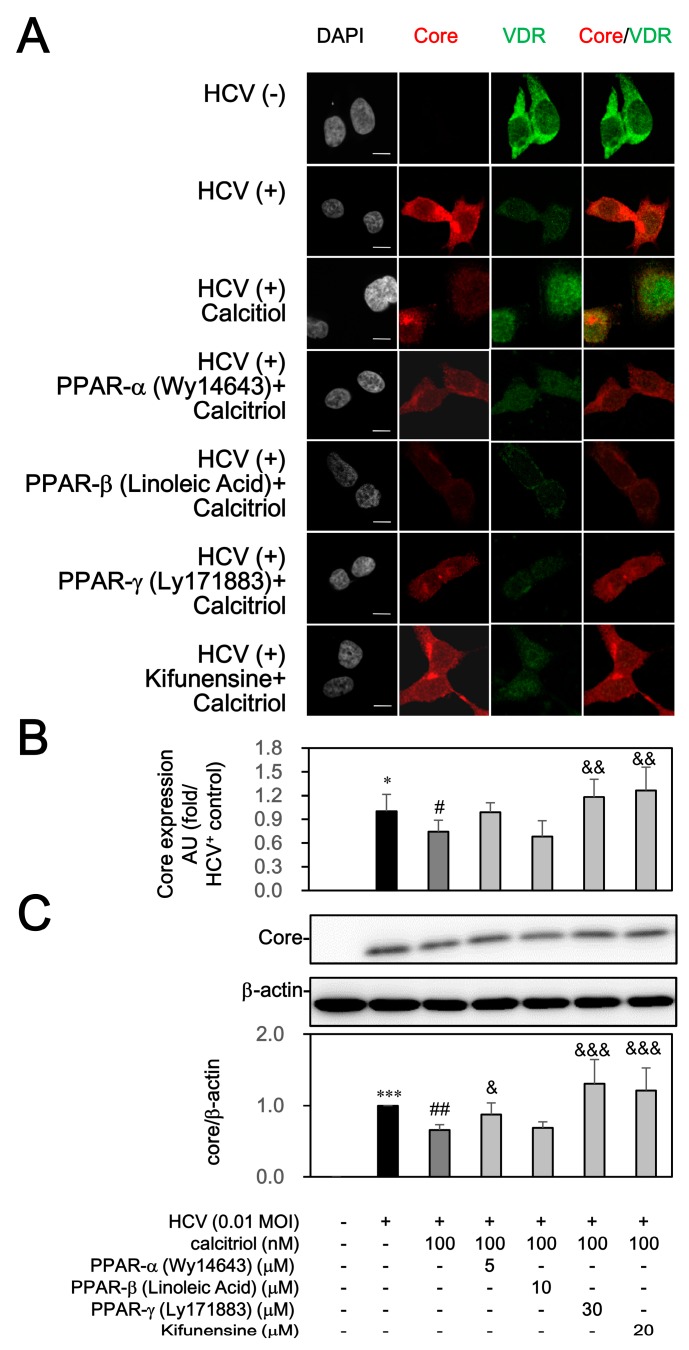
Effects of PPAR stimulators and ERAD inhibitor on calcitriol-mediated the core expression. (**A**) Huh7.5-SEAP cells (1.5 × 10^4^) infected with HCV were treated with 5 μM of Wy14643, 10 μM of linoleic acid, 30 μM of Ly171883, or 20 μM of kifunensine for 1 h ahead of treatments with 100 nM of calcitriol for 6 days in a 24-well plate, fixed with 1% paraformaldehyde, stained with core and VDR antibodies and counterstained with DAPI. After staining, these cells were mounted on glass slides and observed under confocal laser scanning microscope. Scale bar, 10 μM; (**B**) The arbitrary units (AU) of immunofluorescence intensity were assessed using Image J; (**C**) Cell lysates (7 × 10^5^) were collected and the core level was detected by Western blot. Data are expressed as mean ± SD obtained from three individual experiments. * *p* < 0.05 and *** *p* < 0.001 vs. the Huh7.5 cells without HCV infection. ^#^ *p* < 0.05 and ^##^ *p* < 0.01 vs. the HCV-infected Huh7.5-SEAP group. ^&^ *p* < 0.05, ^&&^ *p* < 0.01 and ^&&&^ *p* < 0.001 vs. the calcitriol-treated group. The symbol (–) and (+) referred to the cells without and with HCV infection, respectively.

To clarify whether the PPAR and ERAD could affect the calcitriol-mediated HCV suppression, the HCV-infected cells were exposed to PPAR activators or ERAD inhibitor before calcitriol treatment was also investigated. Wy14643 at 5 µM, linoleic acid at 10 µM and Ly171883 at 30 µM were used to activate PPAR-α, PPAR-β and PPAR-γ, respectively [31]. HCV slightly induced PPRE activity, which was decreased by calcitriol on day 1. These inhibitory effects of calcitriol were reversed by the PPAR-*α* stimulator (Wy14643) and PPAR-γ (Ly171883), whereas PPAR-β (linoleic acid) resulted in a slight but not significant reverse. In addition, an ERAD inhibitor, kifunensine, was used, which, at 5–20 µM, inhibits class 1α-mannosidases to further block the ERAD pathway [32,33]. As compared to various PPAR activators, kifunensine did not affect PPRE activity (Figure 2A). When cells were pretreated with the Ly171883 and kifunensine, the calcitriol-mediated HCV suppression was reversed significantly. However, Wy14643 resulted in a slight reverse on day 6. Kifunensine alone enhanced apparently HCV infection, whereas treatment of PPAR stimulators alone did not result in these results (Figure 2B). Treatments of PPAR stimulators and kifunensine did not induce cytotoxicity (Figure 2C). Consistently, a significantly increased core-positive expression had been quantified in HCV infection, which was reversed by calcitriol treatment (Figure 3A,B). Pretreatment with Ly171883 and kifunensine clearly reversed the calcitriol-mediated inhibition of core expressions (Figure 3A,C).

### 3.3. Calcitriol Blocked NO Release and Lipid Accumulation via PPAR and ERAD

**Figure 4:**
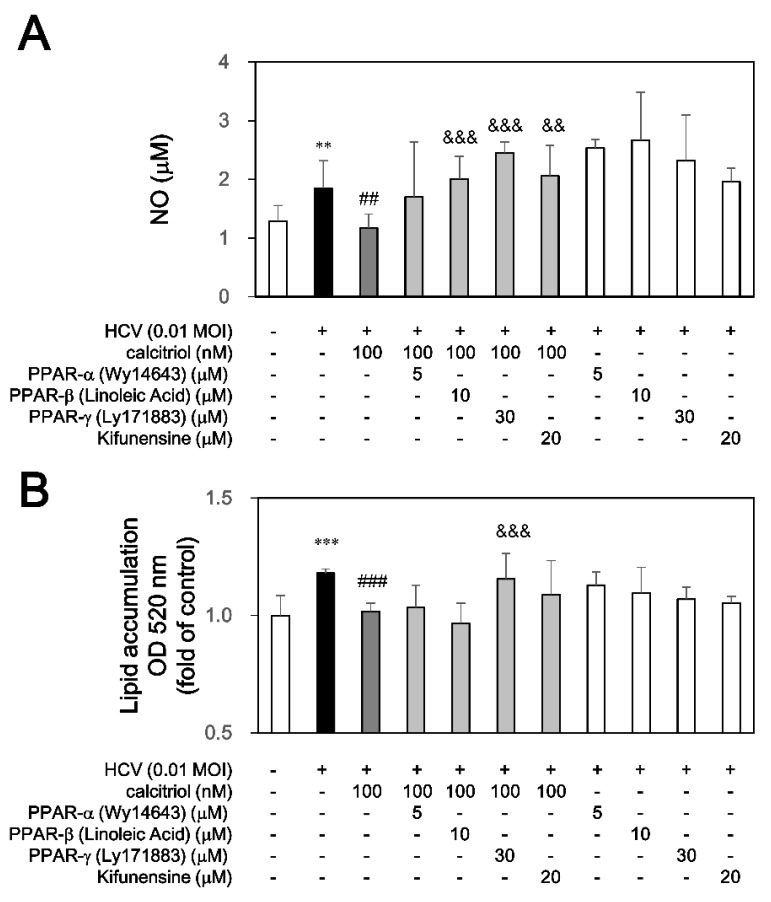
Effects of calcitriol on NO release and lipid accumulation in HCV infection. (**A**) HCV-infected Huh7.5-SEAP cells (2 × 10^4^) were pretreated with 5 μM of Wy14643, 10 μM of linoleic acid, 30 μM of Ly171883, or 20 μM of kifunensine for 1 h before calcitriol treatment in a 24-well plate for 6 days. Cell supernatants were collected for determining NO release by Griess reagent; (**B**) Cells were fixed with formaldehyde, stained with oil red O solution and hematoxylin on day 6, added IGEPAL-CA-630 and then determined by ELISA reader. Data are expressed as mean ± SD obtained from three individual cultures. ** *p* < 0.01 and *** *p* < 0.001 vs. the medium control cells without HCV. ^##^ *p* < 0.01 and ^###^ *p* < 0.001 vs. the HCV-infected cells. ^&&^ *p* < 0.01 and ^&&&^ *p* < 0.001 vs. the calcitriol-treated group. The symbol (–) and (+) referred to the cells without and with HCV infection, respectively.

Vitamin D or calcitriol regulates lipid metabolism [16,17,18,19,20] and prevents nitrative stress [25]. The role of calcitriol in HCV-induced nitrative stress and lipid metabolism was also examined. As expected, calcitriol decreased the HCV-induced NO release. Moreover, pretreatment with linoleic acid, Ly171883 and kifunensine but not Wy14643, counteracted the calcitriol-mediated NO release (Figure 4A). An amount of neutral lipid by oil red O staining was detected in Huh7.5-SEAP cells infected with HCV, which was significantly reduced by treatment of calcitriol. Ly171883 could reverse the calcitriol-mediated inhibition of lipid accumulation, whereas Wy14643, linoleic acid and kifunensine did not affect these results (Figure 4B).

### 3.4. Calcitriol-Induced VDR Activation Was Blocked by PPAR and ERAD Signaling Pathway under HCV Infection

**Figure 5:**
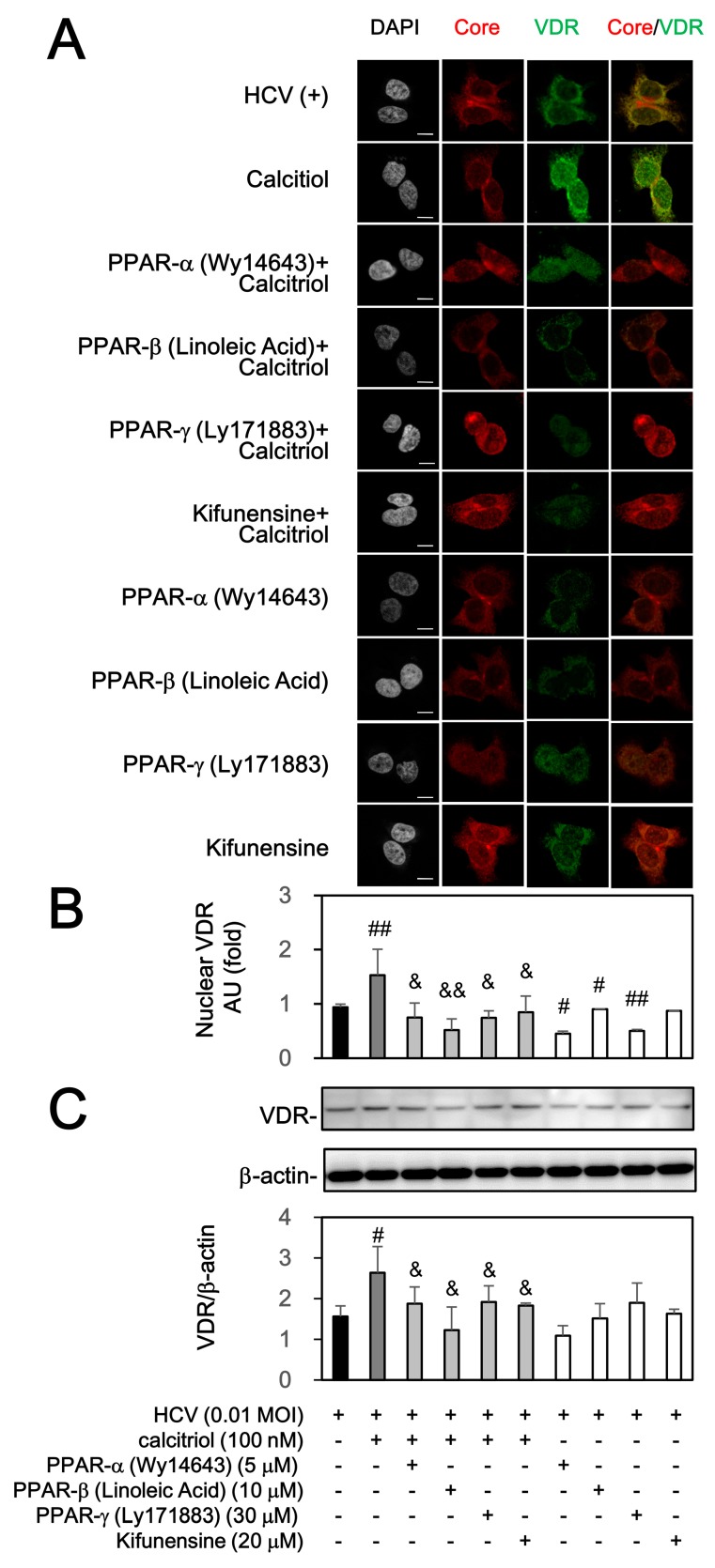
Effects of calcitriol, PPAR stimulators and ERAD inhibitor on expression of VDR. (**A**) HCV-infected Huh7.5-SEAP cells (1.5 × 10^4^) were treated with 5 μM of Wy14643, 10 μM of linoleic acid, 30 μM of Ly171883, or 20 μM of kifunensine for 1 h ahead of treatments with 100 nM of calcitriol for 6 days in a 24-well plate, fixed with 1% paraformaldehyde, stained with core and VDR antibodies and counterstained with DAPI. After staining, these cells were mounted on glass slides and observed under confocal laser scanning microscope. Scale bar, 10 μM; (**B**) The arbitrary units (AU) of immunofluorescence intensity were assessed using Image J; (**C**) Cell lysates (7 × 10^5^) were collected and the VDR level was detected by Western blot. Data are expressed as mean ± SD obtained from three individual experiments. ^#^ *p* < 0.05 and ^##^ *p* < 0.01 vs. compared with the HCV-infected Huh7.5-SEAP group. ^&^ *p* < 0.05 and ^&&^ *p* < 0.01 vs. the calcitriol-treated group. The symbol (–) referred to the cells without HCV infection; the symbol (+) referred to the cells with HCV infection; the symbol (++) referred to the cells with HCV infection and co-treated with drug (e.g., calcitriol, individual PPAR agonist, or ERAD inhibitor); the symbol (+++) referred to the HCV-infected cells pre-incubated with individual PPAR agonist or ERAD inhibitor before calcitriol treatment.

As previously described (Figure 1D), exogenous calcitriol could restore the down-regulation and activate VDR in HCV infection. We further investigated whether the PPAR and ERAD signaling pathways could influence the activation and nuclear translocation of VDR in the presence of calcitriol. The subcellular distribution of VDR was quantified by Image J in order to verify nuclear translocation. Calcitriol could increase by 2.2 ± 0.1 fold in the amount of VDR in the nucleus under HCV infection (Figure 5A,B). As compared with the calcitriol-treated cells, pretreatments of Wy14643, linoleic acid, Ly171883, or kifunensine markedly blocked nuclear translocation of VDR to some extent. Besides, three PPAR activators or kifunensine themselves also decreased the VDR expression (Figure 5A,C). These results indicated that PPAR and ERAD were involved in this process.

### 3.5. Calcitriol Decreased the HCV-Induced apoJ and Grp78 Expression via ERAD

**Figure 6:**
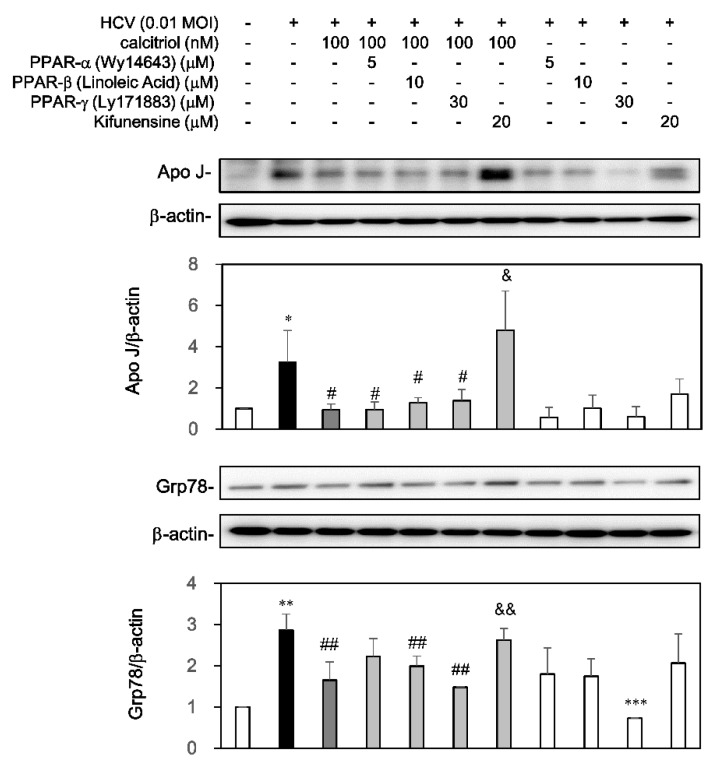
Effects of calcitriol on apoJ and Grp78 in HCV infection. HCV-infected Huh7.5-SEAP cells (7 × 10^5^) were pretreated with 5 μM of Wy14643, 10 μM of linoleic acid, 30 μM of Ly171883, or 20 μM of kifunensine for 1 h before calcitriol treatment in a 6-well plate. Cell lysates were collected at day 6 and the apo J, Grp78 and β-actin levels were detected by Western blot. Data are expressed as mean ± SD obtained from three individual cultures. * *p* < 0.05, ** *p* < 0.01 and *** *p* < 0.001 vs. the medium control cells without HCV. ^#^ *p* < 0.05 and ^##^ *p* < 0.01 vs. the HCV-infected cells. ^&^ *p* < 0.05 and ^&&^ *p* < 0.01 vs. the calcitriol-treated group. The symbol (–) and (+) referred to the cells without and with HCV infection, respectively.

The expression levels of apoJ [34] and Grp78 [35] were induced after HCV infection. Whether calcitriol could block ERAD against HCV infection via apoJ and Grp78 was also examined. Administration of calcitriol could reduce the apoJ level (Figure 6). Pretreatment of kifunensine but not PPAR activators, could reverse the calcitriol-reduced apoJ level. Similarly, the HCV-induced Grp78 expression was inhibited by calcitriol but this expression was reversed markedly by the pretreatment of kifunensine. However, Wy14643 resulted in a slight but not significant reverse (Figure 6).

**Figure 7:**
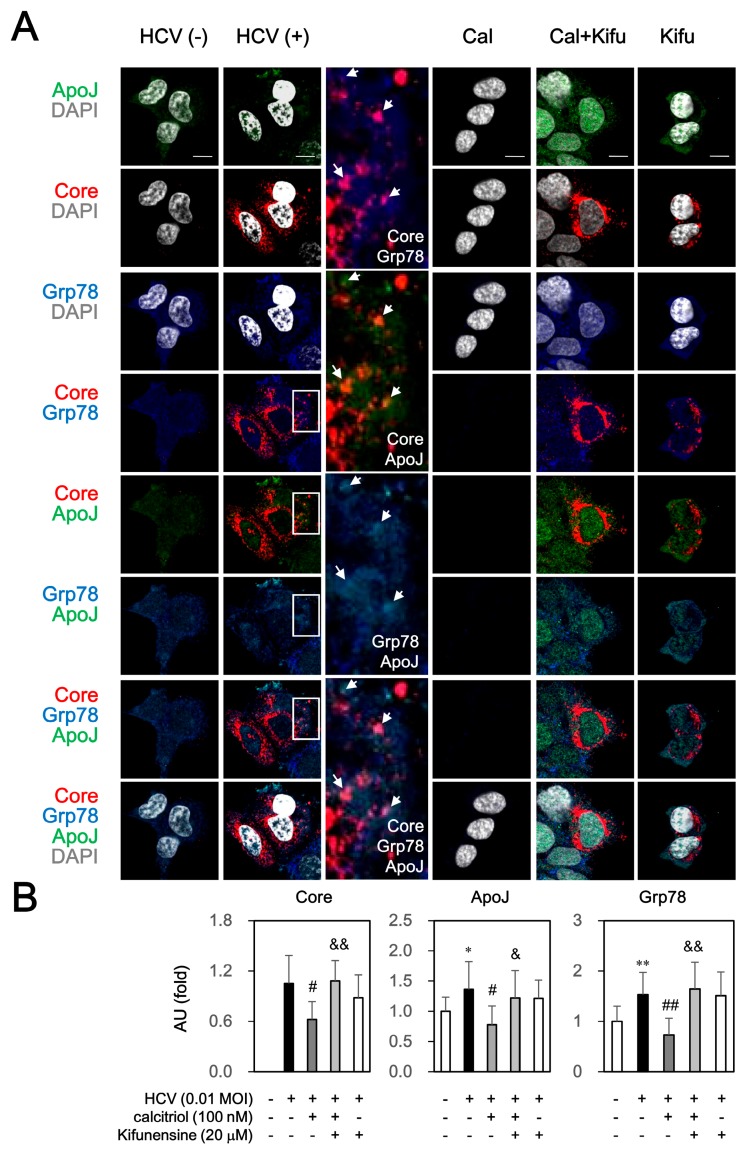
Effect of ERAD inhibitor on calcitriol-mediated protein expressions of core, Grp78 and apoJ under HCV infection. (**A**) HCV-infected Huh7.5-SEAP cells (1.5 × 10^4^) were treated with 20 μM of kifunensine (Kifu) for 1 h ahead of treatments with 100 nM of calcitriol (Cal) for 6 days in a 24-well plate, fixed with 1% paraformaldehyde, stained with the core, Grp78 and apoJ antibodies and counterstained with DAPI. After staining, these cells were mounted on glass slides and observed under confocal laser scanning microscope. White arrow referred to the core protein surrounded by apoJ, co-localization of core and Grp78, or co-localization of apoJ and Grp78. Scale bar, 10 μM; (**B**) The arbitrary units (AU) of immunofluorescence intensity of the core, Grp78 and apoJ were assessed using Image J. Data are expressed as mean ± SD obtained from three independent experiments. * *p* < 0.05 and ** *p* < 0.01 vs. the medium control cells without HCV. ^#^ *p* < 0.05 and ^##^ *p* < 0.01 vs. the HCV-infected Huh7.5-SEAP group. ^&^ *p* < 0.05 and ^&&^ *p* < 0.01 vs. the calcitriol-treated group. The symbol (–) referred to the cells without HCV infection; the symbol (+) referred to the cells with HCV infection; the symbol (++) referred to the cells with HCV infection and co-treated with drug (e.g., calcitriol or kifunensine); the symbol (+++) referred to the HCV-infected cells pre-incubated with kifunensine before calcitriol treatment.

Immunofluorescence analysis showed that HCV core protein was surrounded by apoJ, which was consistent with our previous study [34]. Of note, co-localization of core and Grp78 as well as apoJ and Grp78 was also observed (arrow and amplified in Figure 7). Pretreatment of kifunensine also could reverse the calcitriol-inhibited core, apoJ and Grp78 protein expressions, which localized dispersedly in cytosol and nucleus. These results indicated that calcitriol might interfere ERAD against HCV infection and apoJ and Grp78 participate in this process.

## 4. Discussion

**Figure 8:**
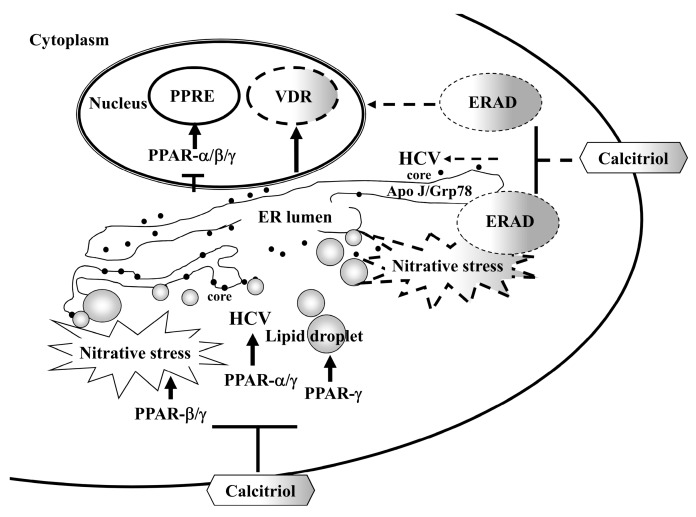
A schematic model of the involvement of PPAR and ERAD in calcitriol-mediated HCV suppression (T-bar). Calcitriol activated VDR but blocked PPAR-α/β/γ activity, meanwhile, it regulated the pathogenicity of HCV, e.g., inhibition of viral infection via PPAR-α/γ, relief of nitrative stress via PPAR-β/γ and reduction of lipid accumulation via PPAR-γ (solid arrow). Alternatively, calcitriol may regulate apoJ and Grp78 through ERAD pathway, which participate in calcitriol-mediated HCV suppression (dotted arrow).

In the present study, calcitriol could directly inhibit HCV. In addition, activation of VDR, interference with ERAD process and blockades of PPAR, lipid accumulation and nitrative stress participated in calcitriol-mediated HCV inhibition. Furthermore, calcitriol hampered the higher protein levels of apoJ and Grp78 against HCV infection (Figure 8).

PPAR-mediated pathways are involved in hepatic inflammation, oxidative stress, lipid metabolism, or cell growth and differentiation [36]. However, several findings regarding the role of PPAR in HCV infection are dissonant: (1) PPAR-α acts to ameliorate steatosis; conversely, the core protein activates PPAR-α which may worsen steatosis via mitochondrial dysfunction [37]; (2) Increased PPAR-γ mRNA expression in liver tissues of chronic hepatitis C patients is associated with HCV-induced steatosis by way of PPAR-γ-up-regulated lipogenic genes that are effectors of lipid accumulation [38]. However, HCV core protein down-regulates PPAR-γ in Huh7 cells, leading to the induction of suppressor of cytokine signaling 7, which is repressed by PPAR-γ [39].

On the other hand, calcitriol modulates lipid metabolism via PPAR-α [18] and -γ [16,17,19,20] and stabilizes the inhibitory VDR proteins [16]. Suppression of PPAR-γ mRNA expressions and down-regulation of the expression of adipogenesis-related genes are involved in this process [17]. Conversely, administration of PPAR-γ agonists reversed the anti-adipogenic effect of VDR [20]. The inhibition of PPAR-α signaling is also connected with VDR [40]. Similarity, calcitriol reduced in part PPRE activity (Figure 2A). Apart from the modulation of VDR activation (Figure 5), PPAR family, especially PPAR-γ, regulated the calcitriol-mediated inhibitory effects, including HCV infection (Figure 2B), NO release (Figure 4A) and lipid accumulation (Figure 4B).

Activation of VDR maintains ER functions [27] and plays a vital role in controlling cholesterol levels [22,41]. Deletion of the macrophage VDR in mice, conversely, results in the activation of ER stress, PPAR-γ signaling and accelerated cholesterol uptake [27]. Administration of calcitriol, however, prevents ER stress [28]. Vitamin D alters apo levels such as apoA1 and apoE [21], otherwise apoJ impedes vitamin D signaling pathway [23,24]. Instead, the modulation of apo levels are essential for HCV production, assembly and secretory machinery [42,43]. ApoJ has been identified as host factor that stabilizes core and NS5A to promote HCV production [34]. On the other hand, the ERAD pathway is a key function of unfolded protein response for the destruction of aberrant proteins. Defects in ERAD accumulates misfolded proteins in the ER and thereby trigger ER stress [44]. Grp78, an ER marker and one of the ERAD components [45], can be induced after HCV infection [35]. HCV infection also activates the ERAD, leading to modulation of virus production [14,46]. Although Grp78 may regulate the stability and retrotranslocation of apoJ under ER stress [26], whether calcitriol blocks ER stress or ERAD against HCV infection remains obscure. Herein, our results showed that calcitriol reduced the HCV-induced apoJ and Grp78 levels (Figure 6 and Figure 7). In addition, blockage of ERAD not only augmented HCV infection (Figure 2 and Figure 3) but also hindered the inhibitory effects of calcitriol, including HCV-induced NO release as well as apoJ and Grp78 expressions (Figure 4A, Figure 6 and Figure 7). Thus, the effect of calcitriol on ERAD in HCV infection might be related to the partial blockade of nitrative stress. Moreover, calcitriol may act as a negative regulator of apoJ and Grp78 and impede the ERAD pathway, which further participated in calcitriol-mediated HCV suppression.

Several studies indicate that the addition of vitamin D2, vitamin D3, 25-hydroxyvitamin D, or calcitriol to IFN-α may suppress HCV production synergistically. However, the above findings regarding vitamin D or its metabolite itself possessing anti-HCV activity appear to be controversial due to some discrepancies, including the different analysis system, culturing time used and drugs administered (before HCV inoculation or co-treated with drug and HCV inoculation), virus strains and MOI [6,7,47,48,49]. Alternatively, we also found that the activation of PPAR and ERAD might interfere the inhibitory effects of calcitriol (Figure 2, Figure 3, Figure 4 and Figure 5). HCV could down-regulate VDR (Figure 1E), which was similar to a previous study that low VDR expression was observed in hepatocytes of chronic hepatitis C subjects [50]. Moreover, HCV could impede the translocation of VDR to nucleus (Figure 1 and Figure 3), whose activation was partially restored after treatment of calcitriol. One previous study has also indicated that VDR may act as a suppressor of IFN-α/Jak/STAT signal pathway [49]. These findings might explain that the partial inhibitory effects of calcitriol on HCV infection occurred in Huh7.5 human hepatoma cells.

Vitamin D deficiency is closely associated with the disease severity of chronic hepatitis C. Anti-inflammatory and immune-modulatory properties may be responsible for logical mechanisms of vitamin D in chronic liver diseases [5]. Nevertheless, the precise mechanisms of calcitriol-mediated inhibition of HCV infection remains obscure. In the present study, we have clarified the notable mechanisms for HCV suppression of calcitriol via the activation of VDR, interference with ERAD process, blockade of PPAR activation, relief of lipid accumulation and reduction of nitrative stress, to further provide an objective strategy for HCV therapy in clinic.

## Abbreviations

apo	apolipoprotein
calcitriol	1,25-dihydroxyvitamin D3
CYP24A1	1,25-dihydroxyvitamin D3 24-hydroxylase
CYP27B1	25-hydroxyvitamin D3 1-α-hydroxylase
DAPI	4,6-diamidino-2-phenylindole
ER	endoplasmic reticulum
ERAD	ER-associated degradation
Grp78/Bip	78 kDa glucose-regulated protein/binding immunoglobulin protein
HCV	hepatitis C virus
IFN-α	interferon-α
MOI	multiplicity of infection
MTS	[3-(4,5-dimethylthiazol-2-yl)-5-(3-carboxymethoxyphenyl)-2-(4-sulfophenyl)-2*H*-tetrazolium]
NO	nitric oxide
NS	non-structural
PPAR	peroxisome proliferator-activated receptor
PPRE	PPAR response elements
RXR SD	retinoid X receptor standard deviation
SEAP	secreted alkaline phosphatase
SVR	sustained virological response
VDR	vitamin D receptor

## References

[1] Han Y.P., Kong M., Zheng S., Ren Y., Zhu L., Shi H., Duan Z. (2013). Vitamin D in liver diseases: From mechanisms to clinical trials. J. Gastroenterol. Hepatol..

[2] Autier P., Boniol M., Pizot C., Mullie P. (2014). Vitamin D status and ill health: A systematic review. Lancet Diabetes Endocrinol..

[3] Iruzubieta P., Terán Á., Crespo J., Fábrega E. (2014). Vitamin D deficiency in chronic liver disease. World J. Hepatol..

[4] Kitson M.T., Roberts S.K. (2012). D-livering the message: The importance of vitamin D status in chronic liver disease. J. Hepatol..

[5] Kitson M.T., Dore G.J., George J., Button P., McCaughan G.W., Crawford D.H., Sievert W., Weltman M.D., Cheng W.S., Roberts S.K. (2013). Vitamin D status does not predict sustained virologic response or fibrosis stage in chronic hepatitis C genotype 1 infection. J. Hepatol..

[6] Gal-Tanamy M., Bachmetov L., Ravid A., Koren R., Erman A., Tur-Kaspa R., Zemel R. (2011). Vitamin D: An innate antiviral agent suppressing hepatitis C virus in human hepatocytes. Hepatology.

[7] Matsumura T., Kato T., Sugiyama N., Tasaka-Fujita M., Murayama A., Masaki T., Wakita T., Imawari M. (2012). 25-Hydroxyvitamin D3 suppresses hepatitis C virus production. Hepatology.

[8] Syed G.H., Amako Y., Siddiqui A. (2010). Hepatitis C virus hijacks host lipid metabolism. Trends Endocrinol. Metab..

[9] Rahman M.A., Dhar D.K., Yamaguchi E., Maruyama S., Sato T., Hayashi H., Ono T., Yamanoi A., Kohno H., Nagasue N. (2001). Coexpression of inducible nitric oxide synthase and COX-2 in hepatocellular carcinoma and surrounding liver: Possible involvement of COX-2 in the angiogenesis of hepatitis C virus-positive cases. Clin. Cancer Res..

[10] De Lucas S., Bartolomé J., Amaro M.J., Carreño V. (2003). Hepatitis C virus core protein transactivates the inducible nitric oxide synthase promoter via NF-kappaB activation. Antivir. Res..

[11] Sheikh M.Y., Choi J., Qadri I., Friedman J.E., Sanyal A.J. (2008). Hepatitis C virus infection: Molecular pathways to metabolic syndrome. Hepatology.

[12] Benali-Furet N.L., Chami M., Houel L., de Giorgi F., Vernejoul F., Lagorce D., Buscail L., Bartenschlager R., Ichas F., Rizzuto R. (2005). Hepatitis C virus core triggers apoptosis in liver cells by inducing ER stress and ER calcium depletion. Oncogene.

[13] Clément S., Pascarella S., Negro F. (2009). Hepatitis C virus infection: Molecular pathways to steatosis, insulin resistance and oxidative stress. Viruses.

[14] Saeed M., Suzuki R., Watanabe N., Masaki T., Tomonaga M., Muhammad A., Kato T., Matsuura Y., Watanabe H., Wakita T. (2011). Role of the endoplasmic reticulum-associated degradation (ERAD) pathway in degradation of hepatitis C virus envelope proteins and production of virus particles. J. Biol. Chem..

[15] Bode J.G., Brenndörfer E.D., Karthe J., Häussinger D. (2009). Interplay between host cell and hepatitis C virus in regulating viral replication. Biol. Chem..

[16] Kong J., Li Y.C. (2006). Molecular mechanism of 1,25-dihydroxyvitamin D3 inhibition of adipogenesis in 3T3-L1 cells. Am. J. Physiol. Endocrinol. Metab..

[17] Zhuang H., Lin Y., Yang G. (2007). Effects of 1,25-dihydroxyvitamin D3 on proliferation and differentiation of porcine preadipocyte in vitro. Chem. Biol. Interact..

[18] Wang W.L., Welsh J., Tenniswood M. (2013). 1,25-Dihydroxyvitamin D3 modulates lipid metabolism in prostate cancer cells through miRNA mediated regulation of PPARA. J. Steroid Biochem. Mol. Biol..

[19] Pînzariu A., Sindilar A., Haliga R., Chelaru L., Mocanu V. (2014). Nutritional factors in transdifferentiation of skeletal muscles to adipocytes. Rev. Med. Chir. Soc. Med. Nat. Iasi.

[20] Salamon H., Bruiners N., Lakehal K., Shi L., Ravi J., Yamaguchi K.D., Pine R., Gennaro M.L. (2014). Cutting edge: Vitamin D regulates lipid metabolism in Mycobacterium tuberculosis infection. J. Immunol..

[21] Lương K.V., Nguyễn L.T. (2012). Theoretical basis of a beneficial role for vitamin D in viral hepatitis. World J. Gastroenterol..

[22] Gonzalez F.J., Moschetta A. (2014). Potential role of the vitamin D receptor in control of cholesterol levels. Gastroenterology.

[23] Shannan B., Seifert M., Leskov K., Boothman D., Pföhler C., Tilgen W., Reichrath J. (2006). Clusterin (CLU) and melanoma growth: CLU is expressed in malignant melanoma and calcitriol modulates expression of CLU in melanoma cell lines in vitro. Anticancer Res..

[24] Shannan B., Seifert M., Boothman D.A., Tilgen W., Reichrath J. (2007). Clusterin over-expression modulates proapoptotic and antiproliferative effects of calcitriol in prostate cancer cells in vitro. J. Steroid Biochem. Mol. Biol..

[25] Garcion E., Wion-Barbot N., Montero-Menei C.N., Berger F., Wion D. (2002). New clues about vitamin D functions in the nervous system. Trends Endocrinol. Metab..

[26] Li N., Zoubeidi A., Beraldi E., Gleave M.E. (2013). GRP78 regulates clusterin stability, retrotranslocation and mitochondrial localization under ER stress in prostate cancer. Oncogene.

[27] Oh J., Riek A.E., Darwech I., Funai K., Shao J., Chin K., Sierra O.L., Carmeliet G., Ostlund R.E., Bernal-Mizrachi C. (2015). Deletion of macrophage vitamin D receptor promotes insulin resistance and monocyte cholesterol transport to accelerate atherosclerosis in mice. Cell Rep..

[28] Haas M.J., Jafri M., Wehmeier K.R., Onstead-Haas L.M., Mooradian A.D. (2016). Inhibition of endoplasmic reticulum stress and oxidative stress by vitamin D in endothelial cells. Free Radic. Biol. Med..

[29] Sun H.Y., Lin C.C., Lee J.C., Wang S.W., Cheng P.N., Wu I.C., Chang T.T., Lai M.D., Shieh D.B., Young K.C. (2013). Very low-density lipoprotein/lipo-viro particles reverse lipoprotein lipase-mediated inhibition of hepatitis C virus infection via apolipoprotein C-III. Gut.

[30] Forman B.M., Tontonoz P., Chen J., Brun R.P., Spiegelman B.M., Evans R.M. (1995). 15-Deoxy-delta 12, 14-prostaglandin J2 is a ligand for the adipocyte determination factor PPAR gamma. Cell.

[31] Kliewer S.A., Forman B.M., Blumberg B., Ong E.S., Borgmeyer U., Mangelsdorf D.J., Umesono K., Evans R.M. (1994). Differential expression and activation of a family of murine peroxisome proliferator-activated receptors. Proc. Natl. Acad. Sci. USA.

[32] Elbein A.D., Tropea J.E., Mitchell M., Kaushal G.P. (1990). Kifunensine, a potent inhibitor of the glycoprotein processing mannosidase I. J. Biol. Chem..

[33] Elbein A.D., Kerbacher J.K., Schwartz C.J., Sprague E.A. (1991). Kifunensine inhibits glycoprotein processing and the function of the modified LDL receptor in endothelial cells. Arch. Biochem. Biophys..

[34] Lin C.C., Tsai P., Sun H.Y., Hsu M.C., Lee J.C., Wu I.C., Tsao C.W., Chang T.T., Young K.C. (2014). Apolipoprotein J, a glucose-upregulated molecular chaperone, stabilizes core and NS5A to promote infectious hepatitis C virus virion production. J. Hepatol..

[35] Yeganeh B., Rezaei Moghadam A., Alizadeh J., Wiechec E., Alavian S.M., Hashemi M., Geramizadeh B., Samali A., Bagheri Lankarani K., Post M. (2015). Hepatitis B and C virus-induced hepatitis: Apoptosis, autophagy and unfolded protein response. World J. Gastroenterol..

[36] Agriesti F., Tataranni T., Ruggieri V., Capitanio N., Piccoli C. (2012). PPARs and HCV-related hepatocarcinoma: A mitochondrial point of view. PPAR Res..

[37] Koike K., Moriya K., Matsuura Y. (2010). Animal models for hepatitis C and related liver disease. Hepatol. Res..

[38] Lima-Cabello E., García-Mediavilla M.V., Miquilena-Colina M.E., Vargas-Castrillón J., Lozano-Rodríguez T., Fernández-Bermejo M., Olcoz J.L., González-Gallego J., García-Monzón C., Sánchez-Campos S. (2011). Enhanced expression of pro-inflammatory mediators and liver X-receptor-regulated lipogenic genes in non-alcoholic fatty liver disease and hepatitis C. Clin. Sci..

[39] Pazienza V., Vinciguerra M., Andriulli A., Mangia A. (2010). Hepatitis C virus core protein genotype 3a increases SOCS-7 expression through PPAR-{gamma} in Huh-7 cells. J. Gen. Virol..

[40] Sakuma T., Miyamoto T., Jiang W., Kakizawa T., Nishio S.I., Suzuki S., Takeda T., Oiwa A., Hashizume K. (2003). Inhibition of peroxisome proliferator-activated receptor alpha signaling by vitamin D receptor. Biochem. Biophys. Res. Commun..

[41] Chow E.C., Magomedova L., Quach H.P., Patel R., Durk M.R., Fan J., Maeng H.J., Irondi K., Anakk S., Moore D.D. (2014). Vitamin D receptor activation down-regulates the small heterodimer partner and increases CYP7A1 to lower cholesterol. Gastroenterology.

[42] Jones D.M., McLauchlan J. (2010). Hepatitis C virus: Assembly and release of virus particles. J. Biol. Chem..

[43] Huang H., Sun F., Owen D.M., Li W., Chen Y., Gale M., Ye J. (2007). Hepatitis C virus production by human hepatocytes dependent on assembly and secretion of very low-density lipoproteins. Proc. Natl. Acad. Sci. USA.

[44] Wang Q., Mora-Jensen H., Weniger M.A., Perez-Galan P., Wolford C., Hai T., Ron D., Chen W., Trenkle W., Wiestner A. (2009). ERAD inhibitors integrate ER stress with an epigenetic mechanism to activate BH3-only protein NOXA in cancer cells. Proc. Natl. Acad. Sci. USA.

[45] Oslowski C.M., Urano F. (2011). Measuring ER stress and the unfolded protein response using mammalian tissue culture system. Methods Enzymol..

[46] Byun H., Gou Y., Zook A., Lozano M.M., Dudley J.P. (2014). ERAD and how viruses exploit it. Front. Microbiol..

[47] Gutierrez J.A., Jones K.A., Flores R., Singhania A., Woelk C.H., Schooley R.T., Wyles D.L. (2014). Vitamin D metabolites inhibit hepatitis C virus and modulate cellular gene expression. J. Virol. Antivir. Res..

[48] Duan X., Guan Y., Li Y., Chen S., Li S., Chen L. (2015). Vitamin D potentiates the inhibitory effect of microRNA-130a in hepatitis c virus replication independent of type I interferon signaling pathway. Mediat. Inflamm..

[49] Lange C.M., Gouttenoire J., Duong F.H., Morikawa K., Heim M.H., Moradpour D. (2014). Vitamin D receptor and Jak-STAT signaling crosstalk results in calcitriol-mediated increase of hepatocellular response to IFN-α. J. Immunol..

[50] Barchetta I., Carotti S., Labbadia G., Gentilucci U.V., Muda A.O., Angelico F., Silecchia G., Leonetti F., Fraioli A., Picardi A. (2012). Liver vitamin D receptor, CYP2R1 and CYP27A1 expression: Relationship with liver histology and vitamin D3 levels in patients with nonalcoholic steatohepatitis or hepatitis C virus. Hepatology.

